# Impact and cost-effectiveness of two interventions to prevent paediatric respiratory syncytial virus disease in Cameroon: a modelling approach

**DOI:** 10.7189/jogh.16.04051

**Published:** 2026-04-30

**Authors:** Farina Leonie Shaaban, Norbert Fuhngwa, Henshaw Mandi, Andrew Clark, Neele Rave, Clint Pecenka, Louis J Bont, Frédéric Debellut, RSV GOLD III – Health Economics Study Group, Louis Bont, Andrew Clark, Frédéric Debellut, Norbert Fuhngwa, Henshaw Mandi, Clint Pecenka, Neele Rave, Farina Leonie Shaaban

**Affiliations:** 1 Department of Paediatrics, University Medical Centre Utrecht, Utrecht, the Netherlands; 2 Triangle Research Foundation Douala, Cameroon; 3 Department of Health Services Research and Policy, London School of Hygiene & Tropical Medicine, London, UK; 4 Center for Vaccine Innovation and Access, PATH, Seattle, Washington, USA; 5 Center for Vaccine Innovation and Access, PATH, Geneva, Switzerland

## Abstract

**Background:**

Preventive interventions for respiratory syncytial virus (RSV) disease are emerging and have been approved for support by Gavi, the Vaccine Alliance. However, their cost-effectiveness is under-researched in low- and middle-income countries, where RSV burden remains the highest. We modelled the potential impact and cost-effectiveness of two interventions for the prevention of RSV in young children in Cameroon, a Gavi-eligible country with a gross domestic product (GDP) per capita of USD 1563 in 2022.

**Methods:**

We used a static proportionate outcomes model to estimate the health and economic burden of RSV with and without a single dose of long-acting infant monoclonal antibody (mAb, nirsevimab) or maternal vaccine (bivalent RSVpreF) administered year-round over the period 2025–2034. We gathered data from the scientific literature, Demographic and Health Surveys, World Health Organization/UNICEF country profiles, clinical trials, and local experts. We calculated RSV cases, clinic visits, hospital admissions, and deaths for each week of age from birth to five years. The primary outcome measure was the incremental cost per disability-adjusted life year (DALY) averted from a government perspective, compared to no intervention. We explored cost-effectiveness over a range of cost-effectiveness thresholds expressed as proportions of the country’s GDP per capita. Due to uncertainty in price per dose and limited country-specific RSV burden data, we ran several deterministic scenarios and performed probabilistic sensitivity analyses.

**Results:**

Between 2025 and 2034, we estimated that at USD 5 per dose, the infant mAb (69% coverage, 77% efficacy, 5 months protection) and maternal vaccine (65% coverage, 69% efficacy, 6 months protection) have a notable impact, averting 2267 (27.3%) and 2226 (26.9%) RSV-related deaths among children under five years old, respectively. In this scenario, the incremental cost was similar for both interventions (USD 500 per DALY averted) when compared separately to no intervention, with >90% probability of being cost-effective at a 0.5 GDP per capita (USD 782) threshold. To be cost-effective in Cameroon, at a 0.1 GDP per capita threshold, both interventions must be priced below USD 2.50.

**Conclusions:**

Both the infant mAb and maternal vaccine have the potential to be impactful and cost-effective in Cameroon and could be affordable if priced appropriately, with support from Gavi.

Acute lower respiratory tract infections (ALRIs) associated with respiratory syncytial virus (RSV) cause substantial morbidity and mortality in children under five years of age. An international pneumonia aetiology study found that RSV was the most frequent cause of severe pneumonia (31% of cases) [1]. Most RSV-related health burden (95% of morbidity and 97% of mortality) in children under five years old is observed in low- and middle-income countries (LMICs), and largely impacts infants aged under six months [2].

In Cameroon, ALRIs are a leading cause of childhood morbidity and mortality. In a hospital-based severe ALRI surveillance study conducted in Yaoundé, Cameroon, in 2011–2013, RSV was detected in 14% of children under five years of age [3]. In a similar setting during the COVID-19 pandemic, 22% of children seeking care for (severe) ALRI tested positive for RSV [4]. Surveillance data from 2018 to 2019 showed 13% average RSV-positivity per season across all age groups, with the highest burden in young children (74% of cases were children under five years of age) [5].

Recent years have seen surges in the development of pharmaceutical RSV preventive interventions [6,7]. Licensed RSV preventive interventions to protect infants present two strategies; first, infant immunisation with a single-dose of long-acting monoclonal antibody (mAb, further modelled as nirsevimab), administered before the RSV season or at birth [8]; second, a bivalent RSVpreF maternal vaccine administered once during the third trimester [9,10]. Both strategies allow for RSV prevention to be incorporated into well-established antenatal care and infant immunisation programmes, potentially strengthening them in the process. Further interventions are also emerging, including RSM01, a mAb in development by the Gates Medical Research Institute, specifically for use in LMICs [11].

The World Health Organization’s (WHO) Strategic Advisory Group of Experts on Immunisation (SAGE) assessed RSV preventive interventions and recommended them for global implementation [10]. National decision-makers will have to assess the value of a potential implementation in their setting. Facing the bulk of the RSV burden, LMICs stand to benefit most from RSV prevention. The health economic evidence needed to inform decision-making efforts is unavailable or scarce for many LMICs, including Cameroon. We aimed to provide an early assessment of the potential cost-effectiveness of RSV prevention in Cameroon, which can be updated as more data become available to guide data-driven decisions.

## METHODS

### Study design

We modelled the potential health impact and cost-effectiveness of introducing the infant mAb and maternal vaccine for RSV prevention in children under five years of age in Cameroon over 10 years. We aggregated the lifetime costs and benefits associated with implementation of RSV interventions among 10 successive birth cohorts (2025–2034), as this allowed us to calculate the potential budget impact and explore changes in parameters over time. While RSV interventions are only expected to be impactful among infants, we included all children under five years of age for completeness. The primary outcome measure was the incremental cost per disability-adjusted life year (DALY) averted (in 2022 USD) from the government perspective, compared to the *status quo* (no pharmaceutical RSV preventive intervention). We performed iterative analyses for several scenarios described in detail below.

In the absence of a recognised national cost-effectiveness threshold (CET) in Cameroon, we calculated the probability that each intervention would be cost-effective over a range of thresholds expressed as proportions of the Cameroonian gross domestic product (GDP) per capita. We assumed a GDP per capita of USD 1563 informed by the World Bank estimate for 2022 [12]. Guided by convention, we used CETs of 0.5 and 1.0 GDP per capita, with an additional conservative CET of 0.1 GDP per capita. This conservative CET corresponded to the midpoint of the range estimated by Ochalek *et al.* for Cameroon (9–12% of GDP per capita) and was chosen because it accounts for opportunity costs of changes in health expenditure [13]. All costs are expressed in 2022 USD, and we used a discount rate of 3% per year for all future costs and benefits.

### Modelling approach

To calculate the potential impact and cost-effectiveness of both interventions, we used the universal vaccine decision-support model (UNIVAC), version 1.7 (London School of Hygiene & Tropical Medicine, London, UK), an Excel-based static proportionate outcomes model with a finely disaggregated age structure (week of age under five years) [14]. This open-access, user-friendly model promotes local ownership and balances model complexity and data paucity, often affecting LMICs. Its setup is described in detail elsewhere [15]. While the core methodology remains consistent with previous studies [15–18], we adjusted input parameters to reflect conditions in Cameroon.

Furthermore, we gathered secondary data from sources including the WHO and the United Nations Children’s Fund (UNICEF), the Demographic and Health Surveys (DHS, 2018, [19]), clinical trials and local experts (**Table 1**). The study team convened with national stakeholders on 11 December 2023 to consult on and validate model input data and build consensus on the modelling strategy. Attending stakeholders were experts from the Expanded Programme on Immunisation Programme, Ministry of Public Health, international and national non-governmental organisations, academia, and national public health institutions in Cameroon. Subsequently, the UNIVAC model was populated to estimate the health and economic burden of RSV, and the impact and costs of introducing the infant mAb or maternal vaccine, expected over the studied period.

**Table 1:** Base case input parameters for evaluating the impact and cost-effectiveness of long-acting infant mAb and maternal vaccine for RSV prevention in Cameroon

	Central value	Uncertainty range	Sources
Incidence rate per 100 000 children under five years old per year*			
Non-severe RSV cases	3740	2750–5079	Li *et al.* [2]
Non-severe RSV visits	2207	1623–2997	DHS 2018 [19]
Severe RSV cases	1400	800–2420	Li *et al.* [2]
Severe RSV visits	826	472–1428	DHS, 2018 [19]
RSV hospital admissions	620	400–940	Li *et al.* [2]
RSV deaths	20	15–27	Li *et al.* [2]
**Non-severe cumulative age-specific burden in %**			Mahmud *et al.* [15]
<1 months	0	NA	
<2 months	2		
<3 months	11		
<6 months	38		
<1 year	66		
<2 years	85		
<3 years	93		
<4 years	97		
<5 years	100		
**Severe cumulative age-specific burden in %**			Mahmud *et al.* [15]
<1 months	4	NA	
<2 months	14		
<3 months	36		
<6 months	60		
<1 year	77		
<2 years	89		
<3 years	95		
<4 years	98		
<5 years	100		
**Disability weights in %**			
Non-severe RSV	5.1	3.2–7.4	GBD 2019 [20]
Severe RSV	13.3	8.8–19.0	GBD 2019 [20]
**Mean duration of illness in days**			
Non-severe RSV	5	3–7	Anderson & Graham [21]
Severe RSV	10	7–14	Anderson & Graham [21]
**Costs of RSV-related care from a governmental perspective in USD**†			
Non-severe RSV disease			
*Outpatient visit cost*	20.93	12.21–33.99	WHO-CHOICE [22], Baral *et al.* [23], Moyes *et al.* [24]‡
Severe RSV disease			
*Outpatient visits cost*	38.72	8.24–125.96	WHO-CHOICE [22]‡
*Hospital admission cost*	180	51.76–902.52	WHO-CHOICE [22], Baral *et al.* [23], Moyes *et al.* [24]‡
**Infant mAb impact**			
Expected coverage (BCG proxy) in %	69	65–87	WUENIC [25], DHS 2018 [19]
Efficacy against severe RSV disease in %	77	49–89	Muller *et al.* [26]
Efficacy against non-severe RSV disease in %§	0.995	NA¶	Muller *et al.* [26]
Duration of protection in months	5	4–6	Muller *et al.* [26]
**Maternal vaccine impact**			
Expected coverage (ANC proxy) in %	65	55–87	DHS 2018 [19], Baral *et al.* [27]
Efficacy against severe RSV disease in %	69	44–84	Kampmann *et al.* [28]
Efficacy against non-severe RSV disease in %§	0.739	0.664–0.794	Kampmann *et al.* [28]
Duration of protection in months	6	3–7	Kampmann *et al.* [28]
**Costs of intervention in USD**†			
Price per dose	5	NA	Assumption
Syringe price per dose	0.028	NA	EPI MoH Cameroon
Safety box price per dose	0.012	NA	EPI MoH Cameroon
International handling cost (% of price per dose)	4	2–6	UNICEF [29]
International delivery cost (% of price per dose)	6	2–15	Debellut *et al.* [30]
Incremental health system cost per dose	2.02	1.01–3.03	ICAN [31]
**Wastage assumptions in % per dose**			
Doses	5	NA	Assumption
Syringes	5	NA	Assumption
Safety boxes	5	NA	Assumption

ANC – antenatal care, BCG – Bacillus Calmette-Guerin, DHS – demographic and health surveys, EPI – expanded programme on immunisation, GBD – global burden of disease study, ICAN – Immunisation Costing Action Network, mAb – monoclonal antibody, MoH – ministry of health, RSV – respiratory syncytial virus, UNICEF – United Nations Children’s Fund, WHO – World Health Organization

*For simplicity, we assumed RSV outcome events to be independent.

†All future costs and benefits are discounted at a 3% discount rate per year. Costs and gross domestic product *per capita* are expressed in 2022 USD.

‡WHO-CHOICE data was supplemented with stakeholder input.

§Expressed as a proportion of efficacy against severe RSV.

¶Interval exceeded the efficacy against severe RSV interval. Assumed equal to the mid value for technical reasons.

### RSV health burden

The UNIVAC model estimates the expected RSV health burden in the absence of preventive interventions (the *status quo*) using population estimates from United Nations population projections [32] to calculate the total number of life-years lived before five years of age, and country-level rates of RSV outcomes. For Cameroon, previous RSV burden studies reported RSV outcomes for specific sub-populations with small sample sizes or were restricted to (urban) facility settings, thus exhibiting low generalizability [3,4,33]. Due to this restriction in national data, we used aggregate estimates of rates of RSV-related cases, hospital admissions, and deaths for countries in the relevant World Bank income classification (*i.e.* lower-middle-income countries) estimated by Li *et al.* [2]. To estimate the rate of RSV-related outpatient visits, we assumed 59% access to pneumonia care as reported in the 2018 Cameroon DHS as a proxy for access to care for RSV ALRI [19]. We assigned RSV burden estimates to each week of age in children under five years using a Burr distribution fitted to UNIVAC default data from Argentina, Kenya, Mozambique, Pakistan, South Africa, and Vietnam, and studies with granular age data [2,15]. We assumed all RSV-related outcome events to be independent. This means that the same child can contribute to multiple outcome events. For example, a child with severe RSV admitted to the hospital following an outpatient visit would contribute to the rate of RSV cases, outpatient visits, and hospital admissions.

### RSV economic burden

We accounted for costs associated with RSV-related outpatient visits and hospital admissions. For non-severe RSV, we estimated average costs for outpatient visits. For severe RSV, we estimated average costs associated with outpatient visits and hospital admissions, independently. Healthcare billing data in Cameroon were scarce, and the available national data for health services delivery costs lacked specificity. We estimated the facility-based healthcare costs associated with RSV infection, using 2021 WHO Choosing Interventions that are Cost-Effective (CHOICE) [22] estimates of health services delivery costs, and estimates of diagnosis and treatment costs provided by national stakeholders based on experience with bronchiolitis and pneumonia care. Since our study explored a government perspective, we omitted all out-of-pocket costs. The diagnosis and treatment costs were adjusted to exclude the average out-of-pocket proportion of healthcare expenditure (68%) in Cameroon [34]. RSV-specific cost of illness data from Malawi and South Africa informed the uncertainty ranges [23,24].

### Intervention coverage

Since neither of these interventions have been introduced in comparable settings, we used proxy data to inform expected coverage for RSV birth-dose and maternal vaccination programmes. For the infant mAb, we assumed birth-dose scheduling for Bacillus Calmette-Guérin (BCG) vaccine as a proxy. We informed BCG coverage by WHO/UNICEF estimates of national immunisation coverage (69% in 2022) [25]. BCG coverage from the 2017 cohort (87%, DHS 2018 [19]) informed the upper bound of the uncertainty range to represent a higher realistic coverage previously achieved for the BCG vaccine in Cameroon [19]. Similarly, we assumed the real-world timeliness (coverage by week of age) estimated for BCG would be a reasonable proxy for the real-world timeliness of an RSV infant mAb. Therefore, we assumed that doses with delayed administration have an impact on RSV disease later in infancy.

Although 71% of recent births in Cameroon were protected against neonatal tetanus [19], maternal tetanus vaccination was a challenging proxy for RSV maternal vaccination due to irregular vaccination schedules depending on parity and tetanus immunisation status preceding pregnancy, as well as obscure timeliness in practice [35]. Instead, we considered attendance at antenatal care (ANC) visits to be an appropriate proxy for achievable RSV maternal vaccine coverage because it represents the potential for access to a skilled healthcare provider within the target gestation window for RSV vaccination (24–36 or 32–36 weeks). We assumed the maternal vaccine could achieve coverage equal to the proportion of pregnant women attending at least four ANC visits (64.9%). In Cameroon, only 41% of first ANC visits in 2018 were during the first trimester [19], thus reducing the chances of attending four ANC visits. To address this potential underestimation, we used the proportion of women attending at least one ANC visit (87%) as the upper bound of the uncertainty range. We also used a more conservative lower bound (55%) based on estimates by Baral *et al.* of maternal vaccine coverage within 24–36 weeks of gestation, accounting for ANC timing [27].

### Intervention efficacy

Based on evidence from phase 3 clinical trials in term and (late) pre-term infants, we used two efficacy estimates for each intervention corresponding to two trial endpoints: RSV-related lower respiratory tract infection (LRTI) hospital admissions, representing severe RSV outcomes, and any medically attended RSV-related LRTI representing non-severe RSV outcomes. We included efficacy against non-severe outcomes as a proportion of efficacy against severe RSV. For the infant mAb, the two efficacy estimates were very close; however, the trial reported a higher lower bound value for efficacy against non-severe RSV (95% confidence interval (CI) = 62–85) than severe RSV (95% CI = 49–89) [26], resulting in a proportion >1 (0.62/0.49). Due to technical limitations, we omitted the CI and used the central-value proportion. We assumed efficacy to be constant over time and fall to zero after the trial observational period, which defined the duration of protection, with an uncertainty range of ±1 month.

### Intervention costs

We estimated the net costs of each intervention as the difference between the total programme costs of the intervention and the averted healthcare costs resulting from immunisation. The programme costs included all costs of providing the intervention from procuring to administering the infant mAb or maternal vaccine. Because the pricing for both interventions is highly uncertain, we assumed equal prices for them (USD 5 per dose for the base-case analysis). RSV interventions were included as part of Gavi, the Vaccine Alliance’s Vaccine Investment Strategy 2018 for the period 2021–2025 [36]. While Cameroon was eligible for Gavi support, at the time of writing, there was no clarity on the modalities and level of support that may be offered by Gavi to eligible countries. We decided to exclude any Gavi support from our analysis to reflect the full potential costs of introducing the infant mAb or maternal vaccine.

### Intervention impact

We applied intervention coverage and efficacy assumptions to estimate age-specific RSV burden with and without each of the RSV preventive interventions. We calculated the lifetime health effects for all RSV disease outcomes (cases, outpatient visits, hospital admissions, and deaths), culminating in DALYs averted, and expressed as their proportional decrease once the intervention parameters are applied (percentage reduction). Similarly, we calculated the economic impact of RSV preventive interventions as the difference in RSV-related healthcare costs for outpatient visits and hospital admissions with and without intervention, from the governmental perspective (healthcare costs averted). In Cameroon, RSV activity has previously been described to persist most of the year with peaks from September to February, and April to July [3,4]. For simplicity, we assumed steady RSV incidence throughout the year and year-round immunisation.

### Scenario and uncertainty analyses

To account for uncertainty in input parameters, we ran deterministic and probabilistic scenario analyses. We performed deterministic scenario analyses adjusting the intervention price and efficacy assumptions:

− Intervention price per dose increased to USD 10 and USD 15.
− Waning efficacy: as described by Mahmud *et al.* [15], we assumed a gamma-fitted efficacy scenario, whereby efficacy declines gradually, approaching zero at 12 months.
− Exploratory analyses: we estimated the price threshold under which both interventions may be considered cost-effective.

While deterministic analyses used the central values of input parameters, probabilistic sensitivity analyses utilised the uncertainty ranges. We assumed simple PERT-Beta distributions informed by the uncertainty ranges and ran 1000 Monte-Carlo simulations for each intervention at a fixed price per dose (USD 5, USD 10, and USD 15). In each simulation, the model probabilistically selected input values contained within the uncertainty ranges and calculated the incremental cost per DALY averted. We present the results from these simulations using cost-effectiveness acceptability curves, showing the probability that each intervention would be cost-effective at a CET equal to 0.1, 0.5, and 1.0 GDP *per capita* (indicated by the point where a chosen CET intersects the curves). These illustrations provide a visual reference for policy makers when evaluating the potential introduction of RSV preventive strategies in Cameroon under uncertainty.

## RESULTS

### Deterministic lifetime effects

Without intervention, we estimated RSV to contribute 204 554 DALYs, including 8291 deaths, in the 10-year cohort of children aged under five years (**Table 2**). In our base-case scenario, both the infant mAb (USD 5 per dose, 69% coverage, 77% efficacy, five months protection) and maternal vaccine (USD 5 per dose, 65% coverage, 69% efficacy, six months protection) exhibited potential for a substantial impact in Cameroon. We estimated the infant mAb to avert 56502 DALYs and 2267 deaths (corresponding to a 27.3% reduction in RSV mortality among children under five years old). Over the modelled period, RSV prevention using infant mAb cost USD 49 million and averted USD 21 million in RSV-associated healthcare costs (net cost = USD 28 million). We estimated that the maternal vaccine averted 55 420 DALYs and 2226 deaths (corresponding to a 26.9% reduction in RSV mortality among children under five years old). With slightly lower programme costs (USD 47 million) and impact on RSV-associated healthcare costs (USD 19 million) than the infant mAb, the maternal vaccine had a similar estimated net cost (USD 28 million).

**Table 2:** Estimated lifetime impact in the first five years of life and costs of RSV preventive interventions compared to no pharmaceutical intervention in Cameroon (2025–2034)*

	No intervention	Infant mAb	Maternal vaccine
**Lifetime costs and effects**			
Non-severe RSV cases	1 854 504	1 504 545	1 619 095
Non-severe RSV visits	1 094 157	887 681	955 266
Severe RSV cases	694 199	504 370	507 793
Severe RSV visits	409 578	297 578	299 598
RSV hospital admissions	307 431	223 364	224 880
RSV deaths	8 291	6023	6064
Total DALYs†	204 554	148 052	149 134
Total healthcare costs in USD†	80 174 333	59 640 271	61 163 510
Total intervention programme costs in USD†	0	48 802 934	46 731 682
**Impact of interventions‡**			
Non-severe RSV cases		349 959	235 409
Non-severe RSV visits		206 476	138 891
Severe RSV cases		189 830	186 406
Severe RSV visits		111 999	109 979
RSV hospital admissions		84 067	82 551
RSV deaths		2267	2226
Proportional reduction, % of non-severe RSV burden		18.9	12.7
Proportional reduction, % of severe RSV burden		27.3	26.9
DALYs averted†		56 502	55 420
Healthcare costs averted in USD†		20 534 062	19 010 823
Net costs of intervention in USD†		28 268 872	27 720 859
**Cost-effectiveness of interventions‡**			
Cost per DALY averted in USD		500	500
GDP *per capita* inUSD		1563	1563
Cost per DALY averted as a proportion of GDP *per capita*, %		31.99	31.99

DALY – disability-adjusted life year, GDP – gross domestic product, mAb – monoclonal antibody, RSV – respiratory syncytial virus, WHO – World Health Organization

*Here, preventive interventions refer to immunisation with infant mAb: USD 5 per dose, 69% coverage, 77% efficacy, five months protection; and maternal vaccination: USD 5 per dose, 65% coverage, 69% efficacy, six months protection.

†All future costs and benefits are discounted at a 3% discount rate per year. Costs and gross domestic product *per capita* are expressed in 2022 USD.

‡No intervention was used as the comparator.

### Cost-effectiveness of RSV immunisation

**Figure 1:**
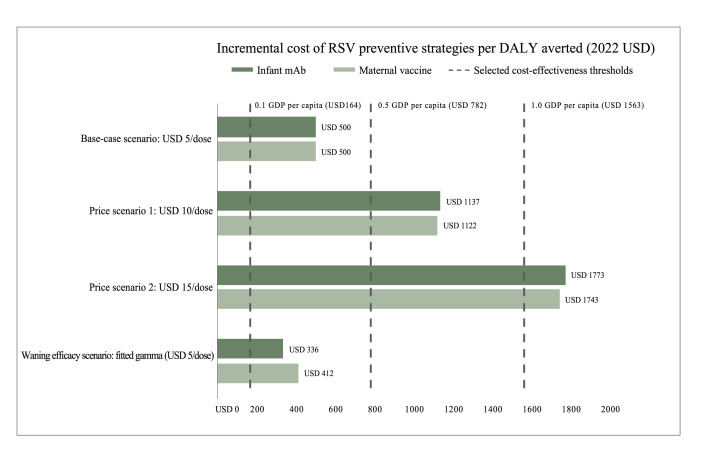
Incremental cost per DALY averted for various scenarios of introducing RSV infant mAb and maternal vaccine in Cameroon over a range of cost-effectiveness thresholds, expressed as proportions of the GDP *per capita*. The base-case scenario assumes USD 5 per dose, and 69% coverage, 77% efficacy, and five months protection for the infant mAb; and 65% coverage, 69% efficacy, and six months protection for the maternal vaccine. In the price scenarios, all else was held constant while the price per dose was increased to USD 10 and USD 15 sequentially. In the waning efficacy scenario, we assumed USD 5 per dose and instantaneous efficacy corresponding to reported cumulative efficacy at trial follow-up time points fitted to a gamma distribution to simulate a more realistic decline in protection, while all else was held constant. DALY – disability-adjusted life year, mAb – monoclonal antibody, GDP – gross domestic product, RSV – respiratory syncytial virus.

Given base-case assumptions, the infant mAb and maternal vaccine were equally cost-effective (USD 500 per DALY averted) when each was compared to the *status quo* (no preventive intervention) (**Figure 1**). This incremental cost corresponded to 32% of the Cameroonian GDP *per capita*, thus considered cost-effective at the conventional CETs of 0.5 and 1.0 GDP *per capita*, but overstepping the conservative 0.1 GDP *per capita* CET. Given base-case assumptions, the infant mAb and maternal vaccine must be priced no higher than USD 2.36 and 2.30 per dose, respectively, to be cost-effective at this conservative CET. The estimated incremental costs reported above were highly sensitive to input parameters. Since the two strategies yielded substantially similar incremental costs, subtle changes in input parameters easily changed the rank order of the two strategies. Therefore, to avoid potentially misleading estimates, we did not model head-to-head comparisons; instead, each RSV immunisation strategy was compared with the *status quo*.

### Scenario and uncertainty analyses

In the price scenarios, the incremental costs per DALY averted were USD 1137 at USD 10 per dose and USD 1773 at USD 15 per dose of the infant mAb; and USD 1122 at USD 10 per dose and USD 1743 at USD 15 per dose of the maternal vaccine. The infant mAb was slightly more costly (less cost-effective) than the maternal vaccine at higher prices per dose (Table S1 in the **Online Supplementary Document**). Incremental costs for both interventions exceeded the 0.1 GPD *per capita* CET at all assumed prices. However, at prices per dose of USD 5 and USD 10, both interventions were cost-effective at the 0.5 and 1.0 GDP *per capita* CETs, respectively. At USD 15 per dose, the incremental costs for both interventions exceeded the 1.0 GDP *per capita* CET.

When we adjusted the base-case scenario to assume waning efficacy, both interventions yielded higher impact, averting 69 384 (infant mAb) and 62 032 (maternal vaccine) DALYs among children under five years old over 10 years (Table S2 in the **Online Supplementary Document**). The incremental cost was USD 336 per DALY averted for the infant mAb and USD 412 per DALY averted for the maternal vaccine. In this scenario, compared to the *status quo*, the infant mAb was more cost-effective than the maternal vaccine, and both interventions were cost-effective at the 0.5 GDP *per capita* CET (**Figure 1**).

**Figure 2:**
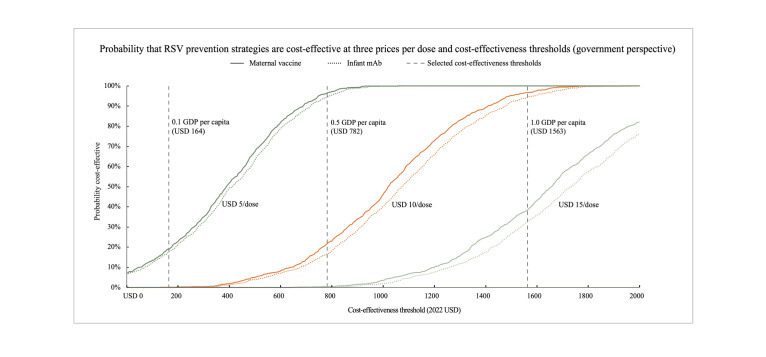
Probability that the infant mAb and maternal vaccine for RSV prevention will be cost-effective in Cameroon at different prices per dose (USD 5, USD 10, and USD 15) over a range of cost-effectiveness thresholds expressed as proportions of the gross domestic product *per capita* (0.1, 0.5, and 1.0). We derived these cost-effectiveness acceptability curves from 1000 Monte-Carlo simulations during probabilistic sensitivity analyses conducted at fixed prices per dose and using the predefined uncertainty ranges to retrieve parameter inputs (**Table 1**). GDP– gross domestic product, mAb – monoclonal antibody, RSV – respiratory syncytial virus.

In probabilistic sensitivity analyses, 1000 Monte Carlo simulations estimated varying incremental costs and benefits of the infant mAb and maternal vaccine, compared to the *status quo*, forming overlapping estimate clouds across price scenarios (Figures S1 and S2 in the **Online Supplementary Document**). The resulting cost-effectiveness acceptability curves suggested that at the 0.5 GDP *per capita* CET (USD 782), the infant mAb and maternal vaccine were >90% likely to be cost-effective if priced USD≤5 per dose. However, cost-effectiveness was unlikely at the conservative 0.1 GDP *per capita* CET, even at USD 5 per dose (probability <20%) for both interventions (**Figure 2**). Appropriate product pricing, with potential Gavi support, could make one or both interventions cost-effective at this conservative threshold.

## DISCUSSION

Here, we provide an early assessment of the potential impact and cost-effectiveness of introducing the RSV infant mAb or maternal vaccine in Cameroon. The presented analyses utilised the most reliable data available for Cameroon, and assumptions were validated by a group of experts. Given our assumptions, both interventions showed potential for substantial impact in Cameroon, possibly averting more than 55 000 DALYs and over a quarter of the RSV-related deaths expected over 10 years among children under five years old. As prices for both interventions remain uncertain and strongly affect cost-effectiveness, we provide independent acceptability estimates for both interventions at several prices and CETs. We assessed incremental costs of each intervention *vs.* the *status quo* at each price per dose, allowing for parallel evaluation of cost-effectiveness. Although we assumed equal prices per dose, it is likely that the infant mAb and maternal vaccine will become available at different costs. Reasons for this include a pricing agreement in place for LMICs for Pfizer’s RSV maternal vaccine and the widely held belief that the mAb cost of goods will be significantly higher than for a maternal vaccine [37].

Additionally, we identified the break-even price per dose at which both interventions are cost-effective at selected CETs. To be cost-effective at the most conservative CET (0.1 GDP *per capita*) [13], and using our parameter assumptions, the infant mAb and maternal vaccine must be priced below USD 2.50 per dose. While break-even prices may aid decision makers in assessing affordability, they should be applied with caution since they are based on model parameters we assumed in deterministic analyses and sensitive to changes in input data.

Cost-effectiveness of the two interventions differed only marginally within pricing scenarios, with the variation most likely driven by subtle differences in assumed efficacy or duration of protection. Cost-effectiveness improved when we assumed the intervention efficacy to be instantaneously high and decline over time, lending preference to the infant mAb over the maternal vaccine (USD 336 *vs.* USD 412 per DALY averted) due to a higher cumulative efficacy. Our analysis remains conservative, as it disregards any strategies to improve affordability beyond product pricing, including Gavi support, to reflect the full price of introducing either intervention. The modalities and level of Gavi support may heavily influence cost-effectiveness, and this analysis should be updated as they become clearer.

The cost-effectiveness of both interventions differs only slightly, and a cost-driven preference depends on the currently unknown price per dose. Other factors not accounted for in this analysis could help more precisely determine the dominant intervention. These include public perception and acceptance of maternal and newborn immunisation, and the level of available infrastructure for implementation, including trained providers, sufficient cold chain logistics, and access to postnatal care and ANC. Country readiness assessments are needed to address these factors. Furthermore, the majority of RSV-related DALYs were attributable to mortality. One might consider an impact-driven approach in the implementation of RSV preventive interventions. For example, targeted prevention efforts in communities where access to care is limited, and thus chances of survival are decreased, will likely considerably enhance our estimated health impact.

Our study has several limitations, mostly resulting from data paucity. We maximised the reliability of proxy data by comparing available sources and selecting data that were most recent, complete, and most applicable to the Cameroonian context, and accounted for uncertainties in probabilistic analyses. We recognise the potential for inaccuracies introduced by using proxy data and recommend follow-up assessments as more data become available. We assumed year-round, unrestricted administration of interventions. Recommendations for seasonal or population-specific administration are likely to change the results presented here, though seasonal approaches require rapid adaptability and may be challenging in low-resource settings. Notably, there are few descriptions of RSV seasonal patterns in Cameroon, and available studies were restricted to short periods and single regions. This gap poses a challenge to a seasonal implementation strategy. We did not factor in discrepancies in equitable access to RSV immunisation. In Cameroon, the populations of zero-dose children and women receiving no ANC remain substantial, with considerable sub-regional differences [19]. Targeted immunisation programmes to reach them may yield higher impact while simultaneously requiring higher programmatic investments. Both infant immunisation and ANC services may stand to benefit from the introduction of RSV immunisation.

Despite international trial recruitment, the efficacy estimates that we used in this study came from trials based mostly in high-income-countries, with lower severe RSV outcome rates compared to LMICs such as Cameroon. It is noteworthy that participants of a controlled clinical trial differ significantly from average LMIC populations. Because pregnant populations in Cameroon have higher rates of complications, including HIV/AIDS, than do trial participants, antibody levels at birth following maternal vaccination may not achieve the reported trial efficacy. Similarly, due to underpowered groups, it is uncertain what level of protection maternal vaccination offers preterm infants. Although mathematical models provide some insights [38], scenarios favouring maternal vaccines may benefit from catch-up programmes with the infant mAb for unprotected high-risk infants [11]. A study in sub-Saharan African countries is evaluating RSV maternal vaccination efficacy and safety in response to a numerical increase in preterm births observed in two upper-middle-income country maternal vaccine trial sites [10]. Our study excluded model inputs on intervention safety in the absence of conclusive data. Understanding the impact of RSV prevention for populations in comparable settings will provide more insight into the potential impact expected in Cameroon.

Lastly, we excluded the impact of indirect effects of RSV prevention, such as changes in healthcare capacity, and opportunity costs of healthcare expenditure as well as excluded RSV-related societal costs, including out-of-pocket healthcare expenditure. This is due to a lack of RSV-specific healthcare utilisation and cost data in Cameroon. We used WHO-CHOICE cost data and expert estimates of costs for pneumonia care as base-case proxies. We also used RSV-specific costing data from the healthcare system perspective in Malawi and South Africa. These estimates varied considerably from the base-case assumption, as well as from each other, and thus informed the uncertainty range. Modelling RSV-related costs from the governmental perspective only underestimated the overall economic impact of RSV prevention. Therefore, ascertaining a measure of household costs for RSV-related care in Cameroon would enable a valuable expansion of the current work.

## CONCLUSIONS

We provide the first assessment of the potential impact and cost-effectiveness of RSV preventive interventions in Cameroon – the infant mAb (nirsevimab) and maternal vaccine (bivalent RSVpreF). Using the UNIVAC decision-support model, our analysis suggests that both interventions could substantially reduce RSV-related morbidity and mortality among children under five years old, potentially preventing over 2000 deaths among children under-five years of age over 10 years, with comparable cost-effectiveness profiles. While these estimates are limited by the scarcity of country-specific data, they provide the best available approximation for the Cameroonian context and address a crucial health economic data gap at a time when large-scale RSV prevention becomes available. Future analyses should update these findings as more (country-specific) data become available, particularly describing RSV-related burden, healthcare utilisation and costs, and product pricing. The use of the UNIVAC decision-support model [14] enables local stakeholders to readily incorporate new evidence and make data-driven decisions on RSV prevention in Cameroon. Decision-makers may evaluate both interventions and assess programmatic feasibility to establish the best-suited strategy for RSV prevention in Cameroon. Apart from factors such as existing infrastructure and public perception, the product price needs to be established. To ensure accessibility to RSV immunisation, these interventions must be priced reasonably. Efforts to accommodate affordability may include tiered pricing, multi-dose vial presentations [11], or licensing of generic products. Gavi support is likely to improve the affordability of RSV preventive interventions.

## Additional material


Online Supplementary Document
