# Supplementary material for: Impact and cost-effectiveness of two interventions to prevent paediatric respiratory syncytial virus disease in Cameroon: a modelling approach
Source: J Glob Health. 2026 Apr 30;16:04051. doi: 10.7189/jogh.16.04051 (PMC13132004; doi:10.7189/jogh.16.04051)
Supplement: Online Supplementary Document [file jogh-16-04051-s001.pdf]

Supplement to:
Shaaban FL, Fuhngwa N, Mandi H, Clark A, Pecenka C, Rave N, Bont LJ, Debellut F. Impact and cost-effectiveness of two interventions to prevent paediatric respiratory syncytial virus disease in Cameroon: a modelling approach. J Glob Health. 2026;16:04051.

Table S1. Estimated costs of implementing RSV preventive interventions (infant mAb: 69% coverage, 77% efficacy, 5 months protection; and maternal vaccine: 65% coverage, 69% efficacy, 6 months protection) in Cameroon (over the period 2025-2034) at USD 10 and USD 15 per dose.

	**Price scenario 1 (USD 10 per dose)**		**Price scenario 2 (USD 15 per dose)**	
	**Infant mAb**	**Maternal vaccine**	**Infant mAb**	**Maternal vaccine**
Total intervention programme costs in USD*	84,769,589	81,171,870	120,736,244	115,612,059
Net costs of intervention in USD*†	64,235,527	62,161,047	100,202,182	96,601,236
**Cost-effectiveness of interventions in USD**				
Cost per DALY averted	1,137	1,122	1,773	1,743
GDP per capita	1,563	1,563	1,563	1,563
Cost per DALY averted, % of GDP *per capita*	72.74	71.79	113.44	111.52

DALY – disability-adjusted life year, GDP – gross domestic product, mAb – monoclonal antibody.
*All future costs and benefits are discounted at a 3% discount rate per year, and costs are presented in 2022 USD
† Net costs = total programme costs – healthcare costs averted

Table S2. Estimated life-time impact in the first five years of life and costs of RSV preventive interventions compared to no pharmaceutical intervention assuming waning efficacy (infant mAb: USD 5 per dose, 69% coverage; and maternal vaccine: USD 5 per dose, 65% coverage; timeframe: 2025-2034).

	Status quo	Infant mAb	Maternal vaccine
**Life-time burden and costs**			
Non-severe RSV outcomes			
Cases	1,854,504	1,397,152	1,602,714
Outpatient visits	1,094,157	824,320	945,601
Severe RSV outcomes			
Cases	694,199	461,052	485,485
Outpatient visits	409,578	272,021	286,436
Hospital admissions	307,431	204,180	215,000
Deaths	8,291	5,506	5,798
Total DALYs*	204,554	135,170	142,521
Total healthcare costs in USD*	80,174,333	54,673,465	59,019,245
Total intervention programme costs in USD*	-	48,802,934	46,731,682
**Intervention impact (comparator: status quo)**			
Non-severe RSV outcomes			
Cases		457,352	251,790
Outpatient visits		269,838	148,556
Severe RSV outcomes			
Cases		233,148	208,715

	Status quo	Infant mAb	Maternal vaccine
Outpatient visits		137,557	123,142
Hospital admissions		103,251	92,431
Deaths		2,784	2,493
Proportional reduction, % of non-severe RSV burden		24.7	13.6
Proportional reduction, % of severe RSV burden		33.6	30.1
DALYs averted*		69,384	62,032
Healthcare costs averted in USD*		25,500,868	21,155,088
Net costs of intervention in USD*†		23,302,066	25,576,594
**Cost-effectiveness of interventions**			
Cost per DALY averted compared to status quo in USD		336	412
2022 GDP per capita in USD		1,563	1,563
Cost per DALY averted, % of GDP *per capita*		21.50	26.36

DALY – disability-adjusted life year, GDP – gross domestic product, mAb – monoclonal antibody, RSV – respiratory syncytial virus.

*All future costs and benefits are discounted at a 3% discount rate per year and costs are presented in 2022 USD

† Net costs = total programme costs – healthcare costs averted

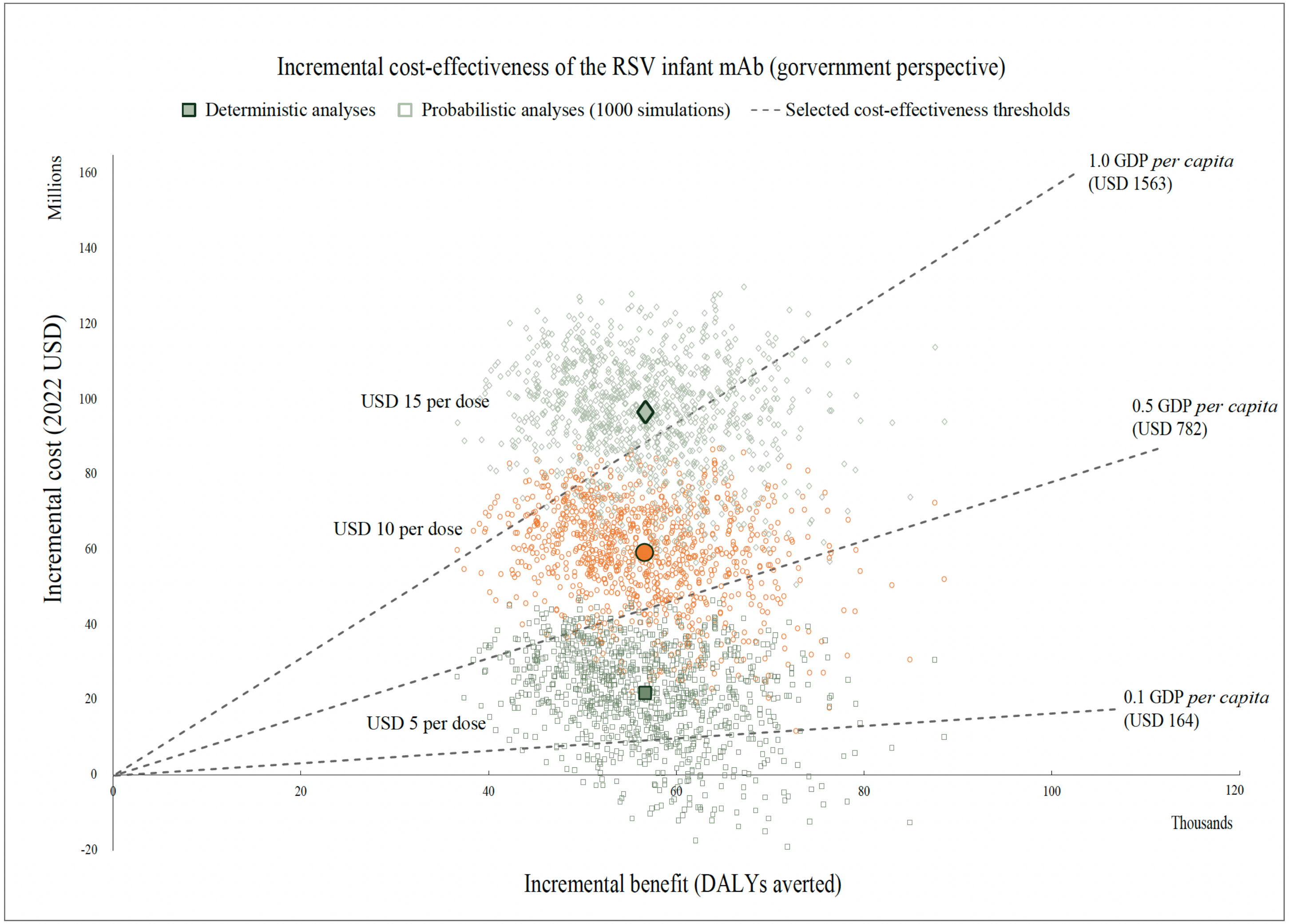

Figure S1. Incremental cost-effectiveness of introducing an RSV infant mAb in Cameroon over the period 2025-2034 for three price scenarios (USD 5, 10, and 15 per dose) across three cost-effectiveness thresholds (0.1, 0.5, and 1.0 times the 2022 GDP *per capita*). DALY – disability-adjusted life year, GDP – gross domestic product, mAb – monoclonal antibody, RSV – respiratory syncytial virus.

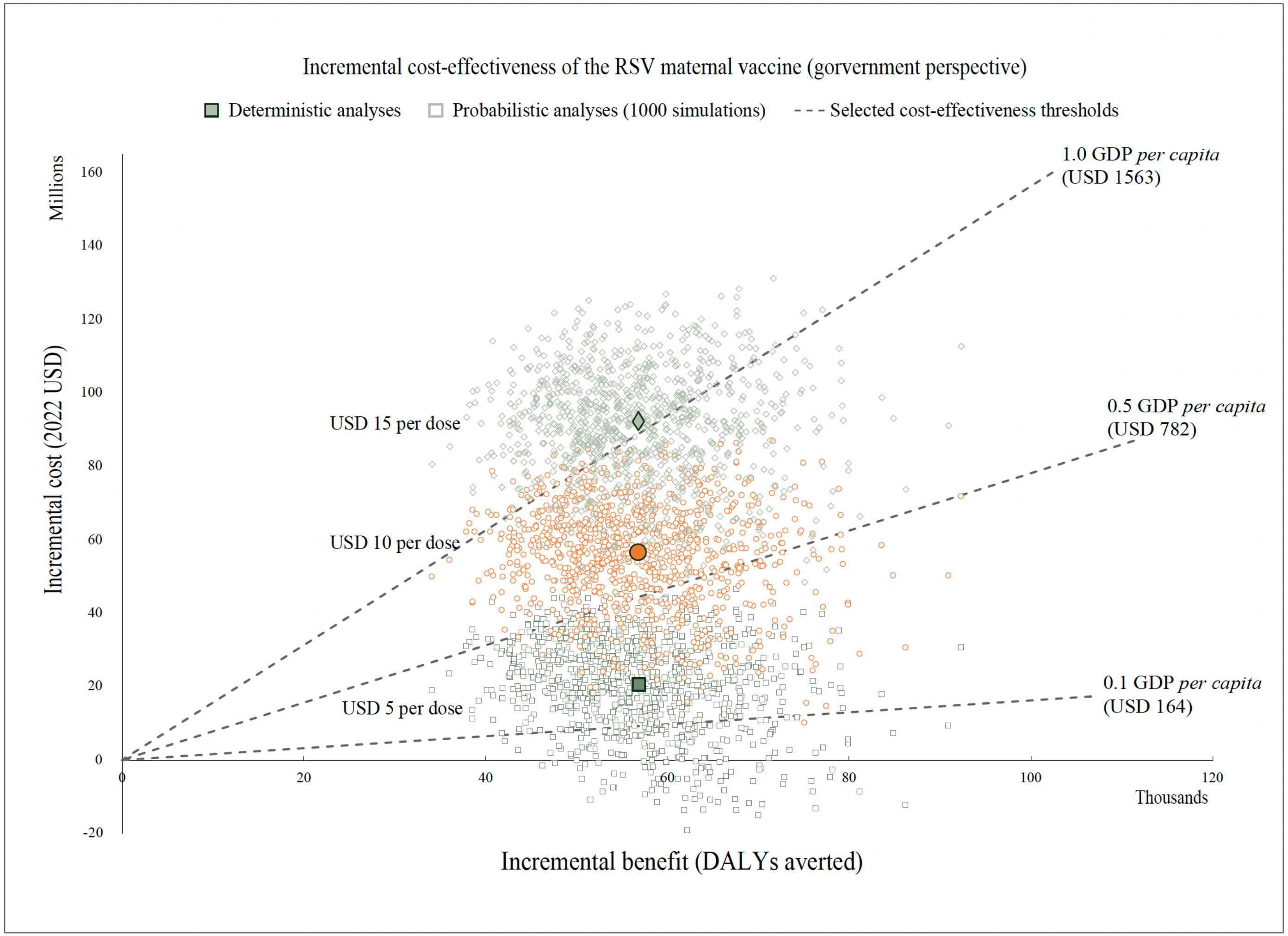

Figure S2. Incremental cost-effectiveness of introducing an RSV maternal vaccine in Cameroon over the period 2025-2034 for three price scenarios (USD 5, 10, and 15 per dose) across three cost-effectiveness thresholds (0.1, 0.5, and 1.0 times the 2022 GDP *per capita*). DALY – disability-adjusted life year, GDP – gross domestic product, RSV – respiratory syncytial virus.

RSV GOLD III – Health Economics Study Group members*

Cameroon:

Frédéric Debellut – Center for Vaccine Innovation and Access, PATH, Geneva, Switzerland
Norbert Fuhngwa – Triangle Research Foundation, Douala, Cameroon
Henshaw Mandi – Triangle Research Foundation, Douala, Cameroon

Ghana:

Rosemary Akuaku – Department of Child Health, Korle Bu Teaching Hospital, Accra, Ghana
Joycelyn Dame – Department of Child Health, University of Ghana Medical School Korle Bu Teaching Hospital, Accra, Ghana
Amma Ekem – Department of Child Health, Korle Bu Teaching Hospital, Accra, Ghana
Bamenla Goka – Department of Child Health, University of Ghana Medical School Korle Bu Teaching Hospital, Accra, Ghana
Ebenezer Ntow – Department of Child Health, Korle Bu Teaching Hospital, Accra, Ghana
Kwabena A. Osman – Department of Child Health, University of Ghana Medical School Korle Bu Teaching Hospital, Accra, Ghana

Mozambique:

Assucênio Chissaque – Instituto Nacional de Saúde, Marracuene district, Maputo, Mozambique; Instituto de Higiene e Medicina Tropical, Universidade Nova de Lisboa, Lisbon, Portugal
Nilsa de Deus – Instituto Nacional de Saúde, Marracuene district, Maputo, Mozambique
Esperança Lourenço Guimarães – Instituto Nacional de Saúde, Marracuene district, Maputo, Mozambique; Instituto de Higiene e Medicina Tropical, Universidade Nova de Lisboa, Lisbon, Portugal
Braiton Maculuve – Ministério da Saúde, Maputo, Mozambique
Elias Manjate – Faculty of Medicine, University Eduardo Mondlane, Maputo, Mozambique
Yara Manjate – Faculty of Medicine, University Eduardo Mondlane, Maputo, Mozambique
Izilda Matimbe – Faculty of Medicine, University Eduardo Mondlane, Maputo, Mozambique
Tufária Mussá – Faculty of Medicine, University Eduardo Mondlane, Maputo, Mozambique
Mirela Pale – Instituto Nacional de Saúde, Marracuene district, Maputo, Mozambique
Cesar Palha – Faculty of Medicine, University Eduardo Mondlane, Maputo, Mozambique

Cristina Sinussene – Faculty of Medicine, University Eduardo Mondlane, Maputo, Mozambique
Farida Zavala – Faculty of Medicine, University Eduardo Mondlane, Maputo, Mozambique

Nepal:
Ram H. Chapagain – Kanti Children's Hospital, Kathmandu, Nepal; Nepal Paediatrics Society, Kathmandu, Nepal
Rita Dhital – Nepal Paediatrics Society, Kathmandu, Nepal
Upendra Dhungana – Public Health Administrator; Ministry of Health and Population. Nepal
Prakash Joshi – Kanti Children's Hospital, Kathmandu, Nepal; Nepal Paediatrics Society, Kathmandu, Nepal
Ranju Karki – Nepal Paediatrics Society, Kathmandu, Nepal
Adita Nepali – Nepal Paediatrics Society, Kathmandu, Nepal
Uttam Paudel – Post Doctorate Researcher (Health Economics), Chulalongkorn University
Arun K. Sharma – Tribhuvan University Teaching Hospital, Institute of Medicine, Kathmandu, Nepal; Nepal Paediatrics Society, Kathmandu, Nepal
Rupesh Shrestha – Tribhuvan University Teaching Hospital, Institute of Medicine, Kathmandu, Nepal
Nirasta Thakili – Nepal Paediatrics Society, Kathmandu, Nepal

Nigeria:
Fadlulai Abdu-Raheem – Department of Paediatrics, Ahmadu Bello University Teaching Hospital, Zaria, Nigeria
Anas Abubakar – Department of Paediatrics, Ahmadu Bello University Teaching Hospital, Zaria, Nigeria
Abdullahi Aminu – Department of Paediatrics, Ahmadu Bello University Teaching Hospital, Zaria, Nigeria
Maria A. Garba – Department of Paediatrics, Ahmadu Bello University Teaching Hospital, Zaria, Nigeria
Fatima J. Giwa – Department of Medical Microbiology, Ahmadu Bello University Teaching Hospital, Zaria, Nigeria
Habiba Lawal – Institute of Child Health, Ahmadu Bello University, Banzazzau, Zaria, Nigeria
Bernsah D. Lawong – Department of Economics, Ahmadu Bello University, Zaria, Nigeria
Abdullahi Musa – Department of Paediatrics, Ahmadu Bello University Teaching Hospital, Zaria, Nigeria
Teddy Naddumba – Center for Vaccine Innovation and Access, PATH, Kampala, Uganda
Aira A. Olorukooba – Department of Paediatrics, Ahmadu Bello University Teaching Hospital, Zaria, Nigeria

Support:
Andrew Clark – Department of Health Services Research and Policy, London School of Hygiene & Tropical Medicine, London, UK
An Nguyen – Center for Vaccine Innovation and Access, PATH, Ho Chi Minh city, Vietnam
Clint Pecenka – Center for Vaccine Innovation and Access, PATH, Seattle, WA, USA

The Netherlands:
Louis J. Bont – University Medical Centre Utrecht, Utrecht, The Netherlands
Neele Rave – University Medical Centre Utrecht, Utrecht, The Netherlands
Farina L. Shaaban – University Medical Centre Utrecht, Utrecht, The Netherlands

*The authors are listed in alphabetical order of their surnames, according to the specific country teams through which they were involved in the study. Team members from University Medical Centre Utrecht, along with supporting staff, were involved at all study sites. A detailed overview of authorship contributions for each country can be found in each respective article.

Text S1. Explanation of authorship change statement

During the manuscript revision process, due to the authors' oversight, the order and designations of corresponding authors needed to be edited. The authors regret any inconvenience this change may have caused.

Author FD was initially listed as the second author in the original submission, and later as the last author. Additionally, author FD was designated as corresponding author instead of author LJB after revision. These changes corrected a clerical error, as FD was intended to be the last and corresponding author from the beginning. The final list of authors is as follows: FLS, NF, HM, AC, NR, CP, LJB, FD; on behalf of the RSV GOLD III – Health Economic Study Group.

All authors have reviewed and agreed to this correction, and we confirm that the requested changes accurately reflect each author’s contributions in accordance with the Journal’s authorship policies.
